# Anal encirclement using polypropylene mesh for high grade complete full-thickness rectal prolapse: A case report

**DOI:** 10.1016/j.ijscr.2019.11.042

**Published:** 2019-11-27

**Authors:** Adeodatus Yuda Handaya, Aditya Rifqi Fauzi, Victor Agastya Pramudya Werdana, Joshua Andrew

**Affiliations:** Digestive Surgery Division, Department of Surgery, Faculty of Medicine, Universitas Gadjah Mada/Dr. Sardjito Hospital, Yogyakarta 55281, Indonesia

**Keywords:** Rectal prolapse, Modified technique, High-risk comorbidity, Elderly patient, Case report

## Abstract

**Introduction:**

Rectal prolapse is defined as protrusion of the rectal wall outside the anus caused by pelvic floor abnormalities. Operative repair is the only definitive treatment. Until now, there is no ideal surgical technique that can be used for all patients.

**Presentation of case:**

Here we report two cases of full-thickness rectal prolapse in elderly patients with high-risk comorbidities. A seventy and seventy-eight-year-old female patients presented with complaints of anal lumps. Their past medical history was significant for arrhythmia, hypertensive heart disease, and pneumonia. The patients then underwent surgical repair with mesh cerclage. The patients were hospitalized for three days after surgery. On days 3, 7, 14, and 6 months after surgery the patients did not complain of any recurrence nor complications.

**Discussion:**

We did a modified anal encirclement surgical repair technique in managing these elderly patients with full-thickness rectal prolapse and high-risk comorbidities using mesh to prevent recurrence.

**Conclusions:**

Anal encirclement technique using mesh can be considered as an alternative procedure for the treatment of full-thickness rectal prolapse in elderly patients with high-risk comorbidities because this procedure is simple, safe, causes fewer postoperative complications, and also can prevent recurrence.

## Highlights

• A modified surgical technique for rectal prolapse:
• Anal encirclement using mesh effective for elderly patients with high risk comorbidities.
• Showed good outcomes.
• No recurrence nor postoperative complications occurred.
• This procedure can be an alternative procedure.

## 1 Introduction

Rectal prolapse or procidentia is a condition in which the rectal wall protrudes through the anus. In complete prolapse, all layers of the rectum undergo circumferential prolapse [[1], [2], [3], [4]]. The incidence of rectal prolapse is not well known. The incidence of complete rectal prolapse in Central Finland reaches 2.5 new cases per 100,000 population per year [2]. Rectal prolapse cases are dominated by women with a ratio of 6:1 compared with men [3]. Many factors are considered to be the cause of rectal prolapses, such as increased intra-abdominal pressure, anal sphincter weakness, and malnutrition. Other causes include rectal inflammation and chronic constipation [5]. The main symptoms of rectal prolapse are constipation and fecal incontinence [6].

The diagnosis of rectal prolapse is clinical, but sometimes defecography, transrectal sonography, and magnetic resonance imaging can help to visualize what cannot be seen in clinical examination. The main therapy for rectal prolapse is surgical repair. The main goal of surgical therapy is the mobilization and fixation of the rectum. Already more than 100 types of surgical techniques have been used to date with various advantages and disadvantages [6,7]. The two approaches usually used to treat rectal prolapse are abdominal and perineal approaches. Perineal procedures are often used in elderly patients or patients with other comorbid conditions, but these procedures have a high recurrence rate, which is around 14–27 % [8].

However, there is still no consensus about the ideal surgical technique for all patients, especially in high-risk surgical patients. Some criteria considered for high-risk surgical patients include patients who are aged >70 years with limited physiological reserve in one or more vital organs [9].

In this report, we aimed to describe a modified anal encirclement procedure in managing two cases of full-thickness rectal prolapse in elderly patients with high-risk comorbidities. This work is an experience of the author as a digestive surgeon. This research work has been reported in line with SCARE 2018 criteria [10].

## 2 Presentation of cases

### 2.1 Case 1

**Fig. 1:**
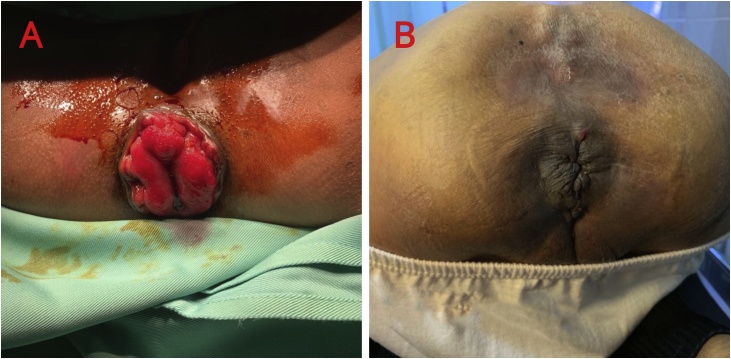
A) Clinical appearance of 4 cm long rectal prolapse in the Case 1. B) Postoperative follow-up at 2 weeks.

A 78-year-old female patient with uterine and rectal prolapse was referred by an obstetrician. The patient had a history of medical treatment due to arrhythmia and hypertensive heart disease. On the physical examination, there was a 4 cm long prolapsed rectum which could still be inserted manually and would come out again when the patient strained (Fig. 1A). The placement of a pessary ring by an obstetrician had been performed to treat her uterine prolapse. Prior to surgery for rectal prolapse repair, the patient was consulted to the anesthesiologist. The anesthesiologist agreed on the need for surgery but assessed the patient with ASA 4, and recommended the procedure be done quickly with regional anesthesia. The cerclage mesh installation was performed and the operation took 30 min. At the time of surgery, the patient is asked to strain to check the function of the sphincter and the function was within normal limits. Postoperatively, the patient was given intravenous 2 × 1 g ceftriaxone injection for three days, chloramphenicol ointment, applied normal saline compress and anal hygiene after defecation. On the third postoperative day, the patient was allowed to go home, wound care was continued at home, and the drug was replaced with 2 × 200 cefixime and 3 × 1 ketorolac orally. At the follow-up on days 3, 7, 14 days, 6, and 12 months after surgery the patient did not complain of any recurrence nor complications (Fig. 1B).

### 2.2 Case 2

**Fig. 2:**
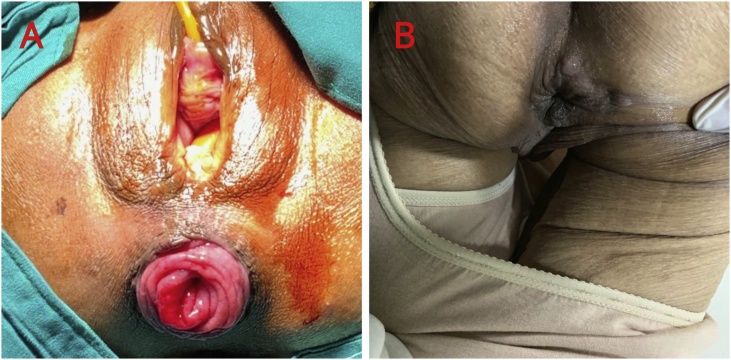
A) Clinical appearance of 3 cm long rectal prolapse. B) Postoperative follow-up at 2 weeks.

A 70-year-old female patient presented to the surgical department with obstipation and anal lumps. The patient had a history of heart and lung treatment due to bronchopneumonia and hypertensive heart disease. On physical examination a 3 cm long rectal prolapse was found that could still be inserted manually and would come out again when the patient strained (Fig. 2A). The patient was consulted to the anesthesiologist for rectal prolapse repair. The anesthesiologist agreed on the need for surgery but assessed the patient with ASA 4, and recommended the procedure be done quickly with regional anesthesia. We proceeded with the mesh cerclage insertion and the operation took 25 min. At the time of the operation, the patient was asked to strain to assess the function of the anal sphincter, and the function was within normal limits. Postoperatively, the patient was given intravenous 2 × 1 g ceftriaxone injection for three days, chloramphenicol ointment, normal saline compress and anal hygiene after defecation. On the third postoperative day, the patient was allowed to discharge, wound care was continued at home, and the drug was replaced with 2 × 200 cefixime and 3 × 1 ketorolac orally. At the follow-up on days 3, 7, 14 days, and 6 months after surgery the patient did not complain of recurrence nor complications (Fig. 2B).

### 2.3 Surgical procedures

1. We prepared the patient in the lithotomy position.
2. We performed the surgical procedure under spinal anesthesia, and then made a 1 cm radial incision in 3, 6, 9, and 12 o’clock (Fig. 3A).

**Fig. 3:**
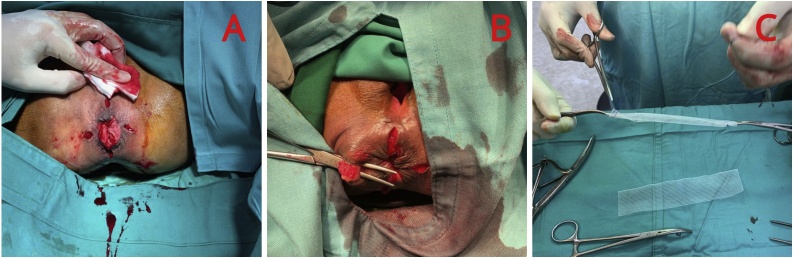
A) Radial incision in 3, 6, 9, and 12 o’clock. B) Circular tunneling at the lateral side in clockwise direction. C) Prolene® mesh preparation.
3. Then, we identified the lateral internal anal sphincter and performed tunneling at the lateral side of it, circularly from 3 to 6 o’clock, 6–9 o’clock, and so on until returning to 3 o’clock direction (Fig. 3B).
4. Then, we prepared Prolene® mesh for about 5–20 cm, and folded it to become layers of 1–10 cm (Fig. 3C), and inserted the mesh circularly starting from 6 to 6 o’clock, in a clockwise direction (Fig. 4A).

**Fig. 4:**
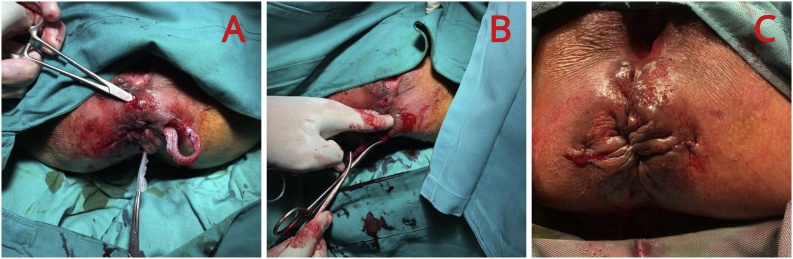
A) Mesh insertion circularly in clockwise direction. B) Evaluation by inserting two fingers to feel the tightness at anal canal. C) subcutaneous skin suturing with Vicryl 2.0 clockwise direction.
5. The evaluation was done by inserting two fingers to feel the tightness of the anal canal to prevent stricture and re-prolapse (Fig. 4B).
6. Finally, we sewed the skin subcutaneously with Vicryl 2.0 in the clockwise incision at 3, 6, 9, and 12 o’clock direction (Fig. 4C).
7. The surgery was completed.

## 3 Discussion

We reported two cases of full-thickness rectal prolapse in patients with high-risk comorbidities treated with a novel surgical procedure by using mesh cerclage. To the best of our knowledge, these cases are the first report of a successful novel procedure in managing rectal prolapse patients with high risk comorbidities. Further study is necessary to confirm and clarify the safety and effectiveness of our procedure.

**Fig. 5:**
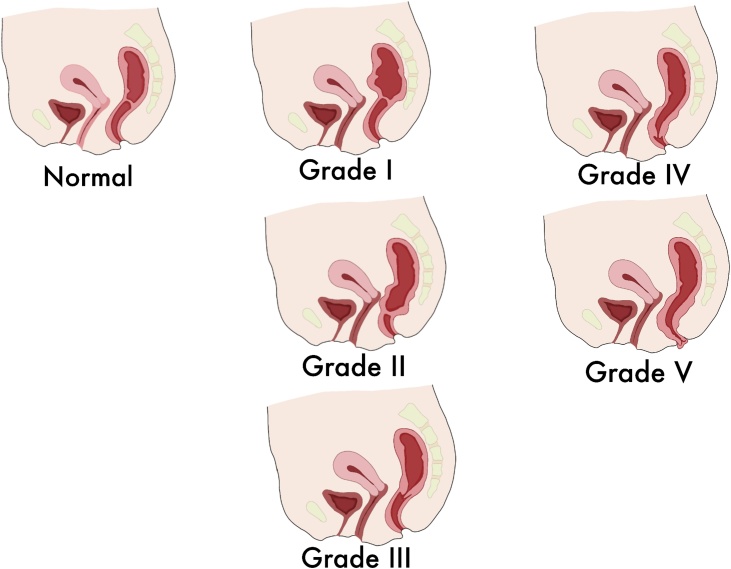
Schematic picture of rectal prolapse grade.

Rectal prolapse can only be entirely corrected with surgical treatment. However, most patients are elderly, and the general performance status is usually poor. Clinically and radiologically, rectal prolapse is graded according to the Oxford Prolapse Grade (Table 1) [7]. Schematic pictures of rectal prolapse grades are shown in Fig. 5.

**Table 1:** Clinical and radiological characteristics of rectal prolapse.

**Internal rectal prolapse**		
**Low grade**	Grade I	Descends to proximal limit of rectocele
	Grade II	Descends into level of rectocele, but not onto anal canal
**High grade**	Grade III	Descends onto anal canal
	Grade IV	Descends into anal canal

**External rectal prolapse**		
	Grade V	Descends through anal canal, protrudes from anus

There are many surgical techniques used to treat prolapse rectum, which are generally divided into two approaches, namely the abdominal and the perineal approaches. The ultimate goals of therapy are to prevent recurrence, restore defecation function, and prevent constipation or incontinence [[11], [12], [13]]. Some authors recommend the use of an abdominal approach in younger rectal prolapse patients, because patients can tolerate general anesthesia, and use the perineal approach in the elderly high-risk population [3].

Our patients are elderly with limited physiological reserve in two vital organs, heart and lungs, so the recommended procedure is transperineally, but another problem to be considered is the high recurrence rate. Anal encirclement using wire has been performed often but has a higher morbidity and recurrence rate [14]. To overcome these challenges, we developed a modified operating technique. In this technique, we used a synthetic material, Prolene® mesh (Johnson Johnson), which was inert when used as a tissue implant and usually used in hernia repair. This mesh is a monofilament first-generation mesh based on polypropylene (PP) systems with a tensile strength of 156.5 N/m and facilitates fibrovascular ingrowth [15]. The goal behind this technique is that the mesh will become a place for new fibrous tissue to grow, so that the anal canal will be tightened permanently and prevent recurrence. A similar technique used by Sainio et al. showed similar outcomes [16].

Finally, although our methods showed good outcomes, our current available evidence does not allow us to conclude whether this procedure is effective for rectal prolapse patients with wider characteristics. Further study should assess in detail the safety and effectiveness of the procedure.

## 4 Conclusions

Based on our preliminary results, the use of mesh material in anal encirclement technique is a safe and simple procedure for treating full-thickness rectal prolapse in patients with high-risk comorbidities, resulting in good outcomes. This modified operative technique can be used as an alternative procedure to treat these kinds of patients. Further larger prospective studies are needed to confirm the effectiveness of this procedure.

## Sources of funding

The authors declare that this study had no funding resource.

## Ethical approval

The informed consent form was declared that patient data or samples will be used for educational or research purposes. Our institutional review board also do not provide an ethical approval in the form of case series.

## Consent

Written informed consent was obtained from the patient for publication of this case report and accompanying images. A copy of the written consent is available for review by the Editor-in-Chief of this journal on request.

## Author contribution

Adeodatus Yuda Handaya conceived the study and approved the final draft. Aditya Rifqi Fauzi, Victor Agastya Pramudya Werdana, and Joshua Andrew Kristianto drafted the manuscript and critically revised the manuscript for important intellectual content. Adeodatus Yuda Handaya, Aditya Rifqi Fauzi, Victor Agastya Pramudya Werdana, and Joshua Andrew Kristianto facilitated all project-related tasks.

## Registration of research studies

Researchregistry5160.

## Guarantor

Adeodatus Yuda Handaya.

## Provenance and peer review

Not commissioned, externally peer-reviewed.

## Declaration of Competing Interest

No potential conflict of interest relevant to this article was reported.
